# Quality Improvement Competencies for Health Care Quality Professionals: Protocol for a Scoping Review

**DOI:** 10.2196/88787

**Published:** 2026-06-04

**Authors:** Nurhayati Shaharuddin, Khalidah Maruan, Divya Nair Narayanan, Normaizira Hamidi, Nurul Izzaty Bahar, Roslina Supadi, Mariyah Mohamad, Samsiah Awang

**Affiliations:** 1 Institute for Health Systems Research National Institutes of Health Ministry of Health Shah Alam, Selangor Malaysia

**Keywords:** quality improvement, competencies, health care quality professionals, scoping review, professional competence

## Abstract

**Background:**

Health care professionals play a crucial role in executing, supporting, and assessing quality improvement programs. Clearly defined competencies in health care quality are essential for optimal performance and serve as the foundation for both quality practice and the design of a capacity development program.

**Objective:**

This study aims to identify and explore the requisite health care quality professional competencies in implementing quality improvement initiatives.

**Methods:**

The study will apply a validated scoping review methodology and the JBI recommendations. An extensive search of the PubMed, Scopus, and Embase databases will be conducted. Gray literature documents will be identified by searching selected Ministry of Health websites in Organization for Economic Co-operation and Development countries and World Health Organization regions with National Quality Policy and Strategy, as well as the Google search engine, using keywords related to quality improvement competencies. The study approach will be reported using PRISMA-ScR (Preferred Reporting Items for Systematic Reviews and Meta-Analyses Extension for Scoping Reviews). Data will be systematically extracted into a matrix using predefined inclusion and exclusion criteria. Analysis will subsequently be conducted using inductive thematic analysis and narrative synthesis.

**Results:**

This scoping review has commenced, and the search strategy has been developed. Preliminary searches conducted in November 2025 through selected electronic databases and gray literature identified 5339 records. Study selection, data extraction, and inductive thematic analysis are scheduled in July 2026 to November 2026. The final findings of this scoping reviews are anticipated to be ready for publication by December 2026. The findings of this study will inform future, larger-scale studies aimed at developing a competency framework for quality improvement among health care quality professionals.

**Conclusions:**

Conducting this scoping review will provide relevant evidence regarding the quality improvement competencies required for health care quality professionals. The results will provide valuable insights to stakeholders in identifying and prioritizing the competencies necessary to enhance the quality improvement skills of health care quality professionals in Malaysia.

**International Registered Report Identifier (IRRID):**

DERR1-10.2196/88787

## Introduction

Quality improvement (QI) in health care involves systematic and continuous efforts to enhance the effectiveness, safety, and efficiency of health care services [1,2] to achieve better patient outcomes, reduce costs, and enhance the overall patient experience [3,4].

To provide high-quality health care services, health care professionals require a certain level of competency, including the knowledge and skills necessary for disease management and QI. For the purpose of the study, health care quality professionals are defined as health care practitioners, such as doctors, nurses or pharmacists, who are formally tasked with executing, coordinating, or overseeing QI work. However, due to the nature of their work, some of them do not serve exclusively as dedicated health care quality professionals but simultaneously continue providing clinical care. As these operational and clinical roles frequently overlap in practice, this scoping review will not strictly differentiate between dedicated quality officers and clinicians. Instead, it will explore the broad spectrum of competencies required by health care professionals undertaking QI responsibilities.

A QI competency is defined as an integrated set of applied knowledge, skills, and attitudes required to perform a given role or responsibility effectively in the quality health care area [5]. Globally, diverse approaches have emerged to define these competencies, each emphasizing key areas critical to effective QI practice. This enables health care quality professionals to gain a comprehensive understanding of their roles in improving health care quality. These competencies are essential and universally applicable across different professions. Categorizing competencies according to proficiency levels will provide a structured framework for guiding capacity development in QI.

Health care quality professionals must possess a wide range of competencies to effectively drive and implement QI initiatives within the health care system. These competencies are typically organized into competency domains, which are defined as broad, overarching categories that group related skills and knowledge together. These domains include leadership, interprofessional collaboration, communication, patient safety, and the ability to manage systemic change [6-10]. Leadership competencies are crucial for guiding teams, influencing organizational practices, and facilitating the adoption of best practices [10,11]. Effective communication and collaboration are equally important, as they enable health care quality professionals to work across multidisciplinary teams, ensuring that diverse perspectives are integrated into care delivery [6,7]. Additionally, competencies in patient safety and risk management are fundamental for identifying potential risks and implementing strategies to reduce harm, ensuring that patient care remains safe, effective, and high quality. Health care quality professionals must also be equipped to drive systemic change by adjusting organizational processes, structures, and policies to foster continuous improvements in health care delivery [8,11].

To ensure health care professionals are well-prepared for these responsibilities, education and training programs must integrate these diverse competencies. Developing strong leadership skills, fostering teamwork, enhancing communication abilities, and prioritizing patient safety are all essential components for professionals to address the challenges of modern health care [8-10]. Moreover, equipping health care quality professionals with the skills to implement systemic changes within organizations is critical for maintaining sustainable improvements in care [12,13]. As health care systems continue to evolve, the integration of these competencies into professional development programs is essential to ensure health care professionals can effectively contribute to the ongoing enhancement of health care quality and safety across diverse health care settings.

In Malaysia, the Ministry of Health (MOH) initiated the Quality Assurance Programme (QAP) in 1985 to enhance the quality, efficiency, and effectiveness of health services provided. The QAP was designed to facilitate an organized and systematic evaluation of quality activities, demonstrating the MOH’s commitment to improving health care standards [14]. Throughout the years, various quality improvement initiatives (QIIs) have been implemented as part of the QAP, including initiatives that track health care performances through disease-specific indicators for both communicable and noncommunicable diseases. In safeguarding patient well-being, efforts targeting patient safety and antimicrobial resistance have been implemented to reduce patient harm. Complementing these efforts are community empowerment initiatives such as Communication for Behavioural Impact (COMBI) for dengue and Healthy Community Builds the Nation (KOSPEN), which encourage community involvement and healthy lifestyle practices. Approach-based initiatives, for example, the Creative and Innovative Circle and lean methodologies, have also been integrated to foster a culture of creativity and innovation in health care delivery [14]. These initiatives are planned, implemented, and monitored by multiple teams of dedicated health care quality professionals at different levels, from the national to the facility level, ensuring comprehensive oversight in achieving their objectives.

However, it is worth noting that most of the QIIs implemented have their capacity strengthening and development plans carried out in silos, with limited collaboration. Selected officers will receive training relevant to their job scope, and participation in these sessions will be formally recognized and rewarded through an individual points system. Currently, there is a lack of explicitly recognized and standardized competencies necessary for health care professionals working in QII units within the health care field in Malaysia [15].

This gap prompted the National Quality Assurance or QI Committee to propose standardized QI competencies essential in building skilled health care professionals working in QII units, enhancing health care delivery, and fostering continuous QI. This initiative aligns with the action plans outlined in the National Policy for Quality in Healthcare, with the ultimate aim of building adequate capacity and capability in QI [15]. Therefore, the proposed scoping review intends to explore and identify the requisite competencies for health care quality professionals working within QII units, serving as a foundational step in this proposed initiative. To ensure a comprehensive capture of existing frameworks, this review will source evidence from both peer-reviewed databases and gray literature, defined as research, reports, conference papers, and official government or organizational documents that are not disseminated through traditional and commercial academic publishing channels [16].

## Methods

### Overview

The proposed scoping review will be conducted in accordance with the framework proposed by Arksey and O’Malley [17] and the JBI methodology for scoping reviews [18]. The methodological framework proposed by Arksey and O’Malley [17] comprises 5 stages for conducting a scoping review. The first 2 stages of this protocol have already commenced, namely, the development of the search strategy and preliminary searches. The remaining stages of the scoping review will be carried out as outlined in this protocol.

### Stage 1: Identifying the Research Question

#### Population, Concept, and Context Framework

The research question was developed using the population, concept, and context framework (Textbox 1).

**Population, concept, and context framework.**

**Component and description**

- Population: only documents that include health care professionals as participants will be included following the review objective. This involves health care professionals from various backgrounds (ie, doctors, nurses, and pharmacists) across all levels (facility, district, state, program, or national) who engage in quality improvement (QI) activities, such as participation in QI projects, oversight or coordination of QI initiatives, and QI management activities. To address varying scopes of practice, this review acknowledges that these healthcare professionals often multitask, performing both clinical duties and QI roles concurrently. Therefore, the review will encompass competencies for this overlapping population rather than strictly differentiating between dedicated healthcare quality professionals and clinicians.
- Concept: the main concept of this scoping review is to identify the QI competencies developed and/or used by health care quality professionals.
- Context: the review will include documents from local and international health care settings. No geographical limitations will be applied for this scoping review. This includes all settings where health care quality professionals provide QI services, such as hospitals, primary care clinics, health state departments, and national health departments.

#### Research Question

What are the QI competencies required by health care quality professionals?

#### Subresearch Question

What are the QI competency domains and subdomains for health care quality professionals that can be identified in the existing literature?

### Stage 2: Identifying Relevant Documents

The search strategy identified documents from 3 databases: PubMed, Scopus, and Embase (Multimedia Appendix 1), which were specifically selected to comprehensively capture clinical, managerial, and international health care policy literature. The main concepts used to develop a customized keyword search were organized around 2 core domains: health care quality professionals and QI competencies. For each database, preliminary searches of document titles and abstracts were conducted to identify key terms related to these 2 core domains. Due to the lack of standardized global nomenclature for health care quality professionals, broad and potentially ambiguous keywords (eg, “quality manager,” “professional competence,” or “continuous improvement”) were deliberately included. This strategy maximized search sensitivity and ensured that relevant literature using alternative or generalized terminology was not excluded. The initial keywords were then adapted to the specific syntax, wildcards, and controlled vocabularies of each database (eg, mapped to Medical Subject Headings [MeSH] terms in PubMed, Emtree in Embase, and using TITLE-ABS-KEY limiters in Scopus). This search strategy was tested and refined as needed to ensure its suitability for the selected databases and keywords. All reviewers evaluated the piloted search strategy and provided feedback for revision, and the proposed search strategy was validated by searching the selected databases. The first 100 search results were reviewed to ensure the validity of the search strategy. The reference lists of all included sources of evidence were screened for additional documents.

This review also included gray literature and related documents that were not available as published documents or in international databases. For example, several official documents such as manuals and reports were available online, with the government official websites serving as useful resources for locating such documents, as only a few published documents exist related to quality competencies of health care professionals. Gray literature was sourced from the targeted MOH websites from the Organization for Economic Co-operation and Development countries, countries that had developed their own National Quality Policy and Strategy based on the World Health Organization regions, and using the Google search engine (Multimedia Appendix 2). The selection of targeted websites was based on the fact that these nations have made significant progress in developing guidelines or strategies to enhance the competence of health care professionals in QI [19,20]. The use of the Google search engine was considered supplementary because not all guidelines or documents have been disseminated through scientific journals, and screening at least the first 200 to 300 results relevant to the study scope was considered [21-23]. The authors acknowledge the Google search engine as a limitation, even though the steps to reduce the filter bubble effect were taken due to the search algorithms’ invisibility. To minimize the potential influence of personalized search results, Google searches were conducted using the incognito or private browsing mode, with no active user login, cleared browsing history, and standardized search settings across reviewers. This is because the technology has been used, hiding the complexity of the search algorithms and not revealing the additional information on which the filtering is based [22,24].

### Stage 3: Document Selection

Stages 1 and 2 are already underway, while stage 3 has yet to commence and will be carried out as described in this protocol. The documents retrieved from the search strategy will be collated and uploaded into Excel, with duplicates removed. Three independent reviewers will screen the titles and abstracts of the documents to identify potentially eligible documents. In case of differences in opinion, discussion will be taken to establish agreement on each document. If consensus is not achieved, a fourth reviewer will be consulted. Three reviewers will independently assess the full texts of the documents against the inclusion and exclusion criteria using piloted screening questions (Table 1). Any disagreements among reviewers will be resolved through discussions or by involving a fourth reviewer. All documents that fail to fulfill the inclusion criteria will be excluded.

**Table 1:** Screening questions.

Question			Action		Reason for exclusion
Screening for title or abstract					
	Is the document related to QI^a^ competencies?	Yes: include Unsure: include No: exclude		Other subjects unrelated to quality health care competencies	
	Is the document in the context of health care settings?	Yes: include Unsure: include No: exclude		Not in the context of quality health care settings	
	Is the document in English?	Yes: include Unsure: include No: exclude		Language	
	Is the full text available?	Yes: include Unsure: include No: exclude		No full text	
	Does the document fit any exclusion criteria?	Yes: exclude No: include		Exclusion criteria	
Screening for full texts					
	Is the document related to QI competencies?	All “no” at this level are excluded		—^b^	
	Is the document in the context of health care settings?	All “no” at this level are excluded		—	
	Does the document fit the type of documents included in this review?	All “no” at this level are excluded		—	
	Does the document fit the exclusion criteria?	If “yes,” exclude		—	

^a^QI: quality improvement.

^b^Not applicable.

The inclusion and exclusion criteria for the selected documents are shown in Table 2 and Textbox 2, respectively. The document selection process will exclude newspaper articles, presentation slides, conference proceedings, social media content, unofficial web pages, comments, editorial papers, and opinion papers, as these types of documents may lack the methodological rigor and empirical data required for this study’s “descriptive-analytic” approach. Review articles and protocols are excluded to avoid duplication of evidence, as this scoping review focuses on mapping primary sources. However, to ensure that relevant competency frameworks and conceptual guidance are not overlooked, the reference lists of relevant review articles will be screened to identify additional eligible primary documents. Additionally, peer-reviewed documents focusing on teaching and learning activities, for example, QI competencies for nursing and medical students, will be excluded as these documents do not show the exact competencies required by health care quality professionals. Only documents published in English will be included because English is the universal language understood by all the reviewers and will facilitate a consistent and coherent analysis by the research team. As this study is not funded, the team will face financial barriers when hiring a translator. Using an online translation service may not accurately reflect the technical meaning of the documents; a limitation acknowledged by the authors. While some targeted regions for gray literature are non-English speaking, only documents available in English will be included. Where English translations of relevant documents are available, they will be incorporated. This approach will allow the study to capture key gray literature while remaining consistent with the English-language inclusion criteria and acknowledging practical limitations of financial and translation resources. No time limit will be applied to the published documents, as the authors aim to include as many documents related to the research question as possible.

The search results and the study inclusion process will be reported in full in the final scoping review and presented in a PRISMA-ScR (Preferred Reporting Items for Systematic Reviews and Meta-Analyses Extension for Scoping Reviews) flow diagram [25].

**Table 2:** Inclusion criteria.

Category	Description	Documents related to any of these keywords or characteristics
Subject	QI^a^ competencies For health care professionals	Any documents on QI competencies for health care professionals
Type and scope of documents	Peer-reviewed and gray literature	Documents describing or reviewing the QI competencies for health care professionals Documents from credible sources, for example, reports, journal articles, publications from government or international agencies, and dissertations Documents that provide examples of the conceptual application of the competencies in the field
Document setting	Health care setting of the document	Any type of health care setting (eg, wards, hospitals, and primary care clinics)
Document version	Updated or not updated	If more than 1 version of the same document exists, the most current version is taken
Year of publication	No restrictions	All relevant publications will be included to maximize coverage of the literature
Language	English	Documents that are published in English

^a^QI: quality improvement.

**Exclusion criteria.**

**Document type**

- Newspaper articles, presentation slides, conference proceedings, social media content, unofficial web pages, comments, editorial papers, and opinion papers

**Document version**

- Document is a draft, summary, or has been replaced by a more recent version

**Full-text availability**

- Documents not available or accessible in full text will be excluded

**Peer-reviewed documents**

- Protocols
- Methodological documents (eg, questionnaire development or validation, sampling or recruitment, and novel analysis approaches) and feasibility studies
- Documents focusing on teaching and learning activities (eg, nursing students, medical students, and trainees)
- Documents that report clinical studies
- Documents not within the health care setting (eg, engineering and higher education)

**Document scope**

- Documents that do not contain substantial content related to the review question
- Documents with a primary focus on teaching and learning activities
- Documents that do not report any framework domains or subdomains used in measuring or assessing a quality improvement competency in health care professionals

### Stage 4: Charting the Data (ie, Extracting)

Data for all documents that meet the inclusion criteria will be extracted using a customized data extraction form. The data extraction template is created to guide the data extraction, including document identifiers such as author, document year, document title, document sources (database or gray literature), origin, aim, study design, target population, and health care setting. In addition to the identifying data, the researchers will focus on extracting the domains and subdomains for the QI competencies (Multimedia Appendix 3). To ensure suitability in capturing the data of interest and establish data extraction consistency, the data extraction form will be piloted on at least 5 to 10 eligible documents. Two independent reviewers will extract data from each included document using the data extraction form, which will be modified and revised as necessary throughout the extraction process. The specific revisions will be outlined in the scoping review. Any reviewer disagreement will be addressed through discussion or by involving an additional reviewer. When necessary (and time permits), the documents’ authors will be contacted to solicit any missing or further data.

### Stage 5: Collecting, Summarizing, and Reporting the Results

Given the current lack of clarity regarding the extent of the data available, it is not feasible to anticipate the most effective approach for organizing and disseminating the study findings. However, the data will be analyzed descriptively to obtain a numerical summary of the included documents. This summary will include information such as the total number of documents, the main objective of each document, the type of document, the target population, the year of publication, the country of origin of the document, and the health care settings where competencies are most commonly found. Data regarding the domains and subdomains of QI competencies will be analyzed using an inductive thematic approach [26]. The unit of analysis will be specific textual segments such as sentences or paragraphs that describe knowledge, skills or attitudes, and competencies related to QI. To ensure rigor during the coding process, 2 reviewers will independently perform line-by-line coding of the extracted text. Any disagreement in coding or theme generation will be resolved through discussions or by involving a third reviewer. Codes with similar characteristics will be grouped to generate initial themes. These themes will capture recurring patterns or common characteristics in the data on the domains and subdomains of QI competencies. Themes will be refined and revised as needed to ensure they accurately represent the relevant QI competency domains and subdomains. This inductive thematic synthesis is a highly iterative process, requiring researchers to continuously move back and forth between the raw data, emerging codes, and overarching themes to build an accurate competency framework. The 12-week duration allocated accommodates this rigorous, cyclical process of coding, reviewing, and refining. Subsequently, the final compilation of themes will be defined and tabulated. A narrative synthesis will complement the diagrammatic or tabulated results, describing how the findings relate to the objective and research questions of the review. The study timeline is described in Table 3.

**Table 3:** Study timeline.

Scoping review steps	Timeline
Stage 1: identifying research question	Completed
Stage 2: identifying relevant studies	Completed
Stage 3: study selection	4-6 weeks
Stage 4: charting the data	4-6 weeks
Stage 5: collecting, summarizing, and reporting the results	12 weeks

### Patient and Public Involvement

Patients and/or the public will not be involved in the design, conduct, reporting, or dissemination of this research.

## Results

A preliminary search was conducted in November 2025 to determine appropriate keywords, subject headings, and search string combination regarding QI competencies among health care quality professionals, yielded 5339 records. Study selection, data extraction, and inductive thematic analysis are scheduled to take place between July 2026 and November 2026. Data collection had not yet begun at the time of manuscript submission. The final findings of this scoping reviews are anticipated to be ready for publication by December 2026. This scoping review is expected to systematically map and synthesize the QI competencies identified in the existing literature as necessary for health care quality professionals. The review will identify and categorize QI competency domains and subdomains, providing an overview of how these competencies have been conceptualized and described across different health care contexts.

The findings are expected to highlight areas of convergence and variation in reported QI competencies. This mapped evidence will offer a structured foundation to inform future research, including the development of a competency framework for health care quality professionals.

## Discussion

### Anticipated Findings

Incorporating recognized QI competencies into the practice of health care quality professionals can lead to several beneficial outcomes. Integrating these competencies may standardize QI practices across health care quality professionals, thereby promoting consistency in health care quality practices. The findings of this analysis, along with the suggested QI competencies, may lead to improved engagement and facilitate collaboration among health care quality professionals and policymakers. These competencies can guide the design of both foundational and ongoing educational programs, supporting the professional development of health care quality professionals. Integrating the identified QI competencies into job descriptions and performance evaluations can provide direct and tangible benefits for both health care outcomes and professional development. These recognized QI competencies may serve as a foundation for developing effective interventions and strategies to enhance QI initiatives within the health care sector. Furthermore, comprehending these essential competencies may empower individuals to assess their readiness to actively participate in QI teams.

This scoping review will not assess the identified QI competencies, nor will it critically evaluate the effectiveness of interventions or strategies aimed at developing or improving those competencies. The review is intended to provide a descriptive overview of the existing evidence on QI competency domains and subdomains among health care quality professionals. Furthermore, as scoping reviews do not include a formal quality appraisal of the included papers, the assurance and credibility of the findings may be limited. Nonetheless, the descriptive overview may still inform future research, policy development, and the design of competency frameworks.

### Strengths and Limitations

A key strength of this review is that it will provide updated evidence on QI competencies from 3 online databases, supplemented by gray literature sources, to facilitate the identification of a comprehensive range of competency areas. Nevertheless, several limitations are anticipated. First, this review will include only publications in English, which may exclude relevant documents in other languages. Second, using specific search terms such as “quality improvement,” “competencies,” and “health care professionals,” primarily within titles and abstracts, may result in missing publications related to QI competencies. The inclusion of gray literature may also be considered a methodological limitation. Although it will enhance comprehensiveness of the review and address publication bias, identifying, locating, and accessing gray literature remains challenging. This is due to its decentralized and nonindexed nature, resulting in a less systematic process of identifying and retrieving gray literature, thus making it more difficult to standardize and reproduce.

## Data Availability

All data will be deposited in the Data Repository, National Institutes of Health, Ministry of Health Malaysia. Requests for data can be made to the Head of the Centre for Biostatistics and Data Repository, National Institutes of Health, Ministry of Health Malaysia, upon reasonable request and with permission from the Director General of Health, Malaysia.

## Appendix

Multimedia Appendix 1 Search strategy.

Multimedia Appendix 2 List of grey literature search.

Multimedia Appendix 3 Data extraction form.

## Abbreviations

COMBI: Communication for Behavioural Impact
MeSH: Medical Subject Headings
MOH: Ministry of Health
PRISMA-SCR: Preferred Reporting Items for Systematic Reviews and Meta-Analyses Extension for Scoping Reviews
QAP: Quality Assurance Programme
QI: quality improvement
QII: quality improvement initiative
