# Supplementary material for: Quality Improvement Competencies for Health Care Quality Professionals: Protocol for a Scoping Review
Source: JMIR Res Protoc. 2026 Jun 4;15:e88787. doi: 10.2196/88787 (PMC13280533; doi:10.2196/88787)
Supplement: Multimedia Appendix 1 [file resprot_v15i1e88787_app1.docx]

Appendix 1: Search strategy

| **Database** | **No** | **Search** |
| --- | --- | --- |
| PubMed | #1 | healthcare professional*[MeSH Terms] OR healthcare personnel[MeSH Terms] OR healthcare provider[MeSH Terms] OR medical professional*[Title/Abstract] OR healthcare practitioner*[Title/Abstract] OR healthcare expert*[Title/Abstract] OR healthcare worker*[Title abstract] or healthcare staff[Title/Abstract] |
|  | #2 | professional competence[MeSH Terms] OR knowledge[MeSH Terms] OR competenc*[Title/Abstract] OR skill*[Title/Abstract] OR capabilit*[Title/Abstract] OR qualification[Title/Abstract] OR capacit*[Title/Abstract] OR expertise*[Title/Abstract] |
|  | #3 | quality improvement*[MeSH Terms] OR quality assurance[MeSH Terms] OR quality management [MeSH Terms] OR QI[Title/Abstract] OR continuous improvement [Title/Abstract] OR quality improv*[Title/Abstract] OR improvement initiative*[Title/Abstract] OR performance improvement*[Title/Abstract] OR qualit*[Title/Abstract] |
|  | #4 | #1 AND #2 AND #3 |
| Scopus | #1 | TITLE-ABS-KEY ( "healthcare professional*”) OR ( TITLE-ABS-KEY ( "healthcare personnel*" ) ) OR ( TITLE-ABS-KEY ( "healthcare provider*" ) ) OR ( TITLE-ABS-KEY ( "medical professional*" ) ) OR ( TITLE-ABS-KEY ( "healthcare practitioner*" ) ) OR ( TITLE-ABS-KEY ( "healthcare expert*" ) ) OR ( TITLE-ABS-KEY ( "healthcare worker*" ) ) OR ( TITLE-ABS-KEY ( "healthcare staff*" ) |
|  | #2 | ( TITLE-ABS-KEY ( "professional competence" ) ) OR ( TITLE-ABS-KEY ( "knowledge" ) ) OR ( TITLE-ABS-KEY ( "competenc*" ) ) OR ( TITLE-ABS-KEY ( "skill*" ) ) OR ( TITLE-ABS-KEY ( "capabilit*" ) ) OR ( TITLE-ABS-KEY ( "qualification" ) ) OR ( TITLE-ABS-KEY ( "capacit*" ) ) OR ( TITLE-ABS-KEY ( "expertise*" ) |
|  | #3 | ( TITLE-ABS-KEY ( "quality improvement*" ) ) OR ( TITLE-ABS-KEY ( "quality assurance" ) ) OR ( TITLE-ABS-KEY ( "quality management*" ) ) OR ( TITLE-ABS-KEY ( "qi*" ) ) OR ( TITLE-ABS-KEY ( "continuous improvement" ) ) OR ( TITLE-ABS-KEY ( "quality improv*" ) ) OR ( TITLE-ABS-KEY ( "improvement initiative*" ) ) OR ( TITLE-ABS-KEY ( "performance improvement*" ) ) OR ( TITLE-ABS-KEY ( "qualit*" ) |
|  | #4 | #1 AND #2 AND #3 |
| Embase | #1 | ('health care personnel')/br OR (("healthcare professional"):ab,ti) OR (("healthcare provider*"):ab,ti) OR (("medical professional*"):ab,ti) OR (("healthcare practitioner*"):ab,ti) OR (("healthcare expert*"):ab,ti) OR (("healthcare worker*"):ab,ti) OR (("healthcare staff*"):ab,ti) |
|  | #2 | 'competence'/exp OR competence OR 'professional competence':ab,ti OR knowledge:ab,ti OR skill:ab,ti OR capabilit*:ab,ti OR qualification:ab,ti OR capacit*:ab,ti OR expertise*:ab,ti |
|  | #3 | 'total quality management'/exp OR 'total quality management' OR 'quality improvement*':ab,ti OR 'quality assurance':ab,ti OR 'quality management':ab,ti OR 'qi*':ab,ti OR 'continuous improvement':ab,ti OR 'quality improv*':ab,ti OR 'improvement initiative*':ab,ti OR 'performance improvement*':ab,ti OR 'qualit*':ab,ti |
|  | #4 | #1 AND #2 AND #3 |
