# Supplementary material for: Quality Improvement Competencies for Health Care Quality Professionals: Protocol for a Scoping Review
Source: JMIR Res Protoc. 2026 Jun 4;15:e88787. doi: 10.2196/88787 (PMC13280533; doi:10.2196/88787)
Supplement: Multimedia Appendix 2 [file resprot_v15i1e88787_app2.docx]

Appendix 2: List of grey literature search

| **OECD Countries** | **Organisation** | **Website** |
| --- | --- | --- |
| Australia | Australian Government, Department of Health and Aged Care | [www.health.gov.au](http://www.health.gov.au/) |
|  | Australian Commission on Safety and Quality in Health Care (ACSQHC) | <https://www.safetyandquality.gov.au/> |
| Austria | Federal Ministry Republic of Austria: Social Affairs, Health, Care and Consumer Protection | <https://www.sozialministerium.at/en.html> |
| Belgium | federal public service: Health, Food, Chain Safety and Environment | <https://www.health.belgium.be/en/health> |
| Canada | Canadian Institute for Health Information | <https://www.cihi.ca/en> |
|  | Health Canada | <https://www.canada.ca/en/services/health.html> |
| Chile | Chile Ministry of Health | <https://www.minsal.cl/> |
| Czech Republic | Ministry of Health | <https://www.mzcr.cz/> |
| Denmark | Ministry of the Interior and Health | <https://sum.dk/> |
| Estonia | Health Board | <https://www.sm.ee/en> |
| Finland | Ministry of Social Affairs and Health | <https://stm.fi/en/frontpage> |
| France | Ministry of Social Affairs and Health | <https://solidarites-sante.gouv.fr/> |
| Germany | Federal Ministry of Health (Germany) | <https://www.bundesgesundheitsministerium.de/en.html> |
|  | Robert Koch Institute | <https://www.rki.de/EN/Home/homepage_node.html> |
| Greece | Ministry of Health | <https://www.moh.gov.gr/> |
| Hungary | Ministry of Human Capacities | <https://www.kormany.hu/en/ministry-of-human-resources> |
| Iceland | Ministry of Health | <https://www.government.is/ministries/ministry-of-health/> |
| Ireland | Department of Health | <https://www.gov.ie/en/organisation/department-of-health/> |
| Italy | Ministry of Health | <http://www.salute.gov.it/> |
| Japan | Ministry of Health, Labour and Welfare | <https://www.mhlw.go.jp/english/> |
| Korea | Ministry of Health and Welfare | <https://www.mohw.go.kr/eng/> |
| Latvia | Ministry of Health of the Republic of Latvia | <http://www.vm.gov.lv/en/> |
| Lithuania | Ministry of Health of The Republic of Lithuania | <http://sam.lrv.lt/en/> |
| Luxembourg | Ministry of Health | <http://sante.public.lu/> |
| Mexico | Secretariat of health | <https://www.globalgiving.org/locations/mexico/> |
| Netherlands | Dutch life science | <http://www.dutchlifescience.com/nl/portal/result_companies.php> |
| New Zealand | Ministry of Health | <https://www.health.govt.nz/> |
|  | Health Quality & Safety Commission | <https://www.hqsc.govt.nz/our-data/health-quality-and-safety-indicators/> |
| Norway | Ministry of Health and Care Services | <https://www.regjeringen.no/en/dep/hod/id421/> |
| Poland | Ministry of Health | <https://www.gov.pl/web/zdrowie> |
| Slovak Republic | Ministry of Health of Slovak Republic | <https://www.health.gov.sk/Index.aspx> |
| Slovenia | Ministry of Health | <https://www.gov.si/podrocja/zdravje/> |
| Spain | Ministry of Health, Consumer Affairs and Social Welfare | <https://www.mscbs.gob.es/en/home.htm> |
| Sweden | Ministry of Health and Social Affairs | <https://www.government.se/government-of-sweden/ministry-of-health-and-social-affairs/> |
| Switzerland | Federal Office of Public Health | <https://www.bag.admin.ch/bag/de/home.html> |
| United Kingdom | Department Health and Social Care | <https://www.gov.uk/government/organisations/department-of-health-and-social-care> |
|  | NHS England | <https://www.england.nhs.uk/> |
|  |  | <https://www.england.nhs.uk/publication/> |
|  | GOV.UK | <https://www.gov.uk/> |
|  | National Institute for Health and Care Excellence | <https://www.nice.org.uk/> |
| United States of America | Department of Health & Human Services | <https://www.hhs.gov/> |
|  | AHRQ | <https://www.ahrq.gov/> |
|  | National Committee for Quality Assurance | <https://www.ncqa.org/> |
|  | National Quality Forum | <https://www.qualityforum.org/Home.aspx> |

| **NQPS/ WHO Region** | **Organisation** | **Website** |
| --- | --- | --- |
| Burundi | Minister of Public Health | <http://minisante.bi/> |
| Ethiopia | Ministry of Health | <https://www.moh.gov.et/site/> |
| Ghana | Ministry of Health | <https://www.moh.gov.gh/> |
|  | Ghana Health Services | <https://ghs.gov.gh/> |
| Guinea | Ministry of Health | <https://sante.gov.gn/> |
| Kenya | Ministry of Health | <https://www.health.go.ke/> |
| Liberia | Ministry of Health and Social Welfare | <http://moh.gov.lr/> |
| Mozambique | Ministry of Health | <https://www.misau.gov.mz/> |
| Namibia | Ministry of Health Social Services | <https://mhss.gov.na/> |
| Senegal | Ministry of Health and Social Action | <https://www.sante.gouv.sn/> |
| Sierra Leone | Ministry of health and sanitation | <https://mohs.gov.sl/> |
| South Africa | National Department of Health | <https://www.health.gov.za/> |
| South Sudan | Ministry of Health | <https://moh.gov.ss/> |
| Sudan | National Ministry of Health | [http://http//www/fmoh.gov.sd](http://http/www/fmoh.gov.sd) |
| Tanzania | Ministry of Health | <https://www.moh.go.tz/> |
| Uganda | Ministry of Health | <https://www.health.go.ug/> |
| Zambia | Ministry of Health | <https://www.moh.gov.zm/> |
| Zimbabwe | Ministry of health and child care | <http://www.mohcc.gov.zw/> |
| Afghanistan | Ministry of Public Health | <https://moph.gov.af/en> |
| Qatar | Ministry of Public Health | <https://www.moph.gov.qa/english/Pages/default.aspx> |
| Scotland | Health and Social care | <https://www.gov.scot/health-and-social-care/> |
| Colombia | Ministry of Health and Social Protection of Colombia | <https://www.minsalud.gov.co/English/Paginas/inicio.aspx> |
| Honduras | Ministry of Health | <https://www.salud.gob.hn/site/> |
| Paraguay | Ministry of Public Health and Social Welfare | <https://www.mspbs.gov.py/index.php> |
| Peru | Ministry of Health | <https://www.gob.pe/minsa> |
| Cambodia | Ministry of Health | <http://moh.gov.kh/?lang=en> |
| Indonesia | Ministry of Health Republic of Indonesia | <https://www.kemkes.go.id/> |
| Nepal | Ministry of health and population | <https://mohp.gov.np/en> |
| Sri Lanka | Ministry of Health | <http://www.health.gov.lk/moh_final/english/> |
| Thailand | Ministry of Health | <http://www.moph.go.th/> |
| Timor Leste | Ministry of Health | <http://www.ms.gov.tl/en> |
| Kiribati | Ministry of Health & Medical Services | <https://mhms.gov.ki/> |
| Malaysia | Ministry of Health | <https://www.moh.gov.my/> |

| **Website** | **Search** | **URL** |
| --- | --- | --- |
| Google Search | healthcare professional AND quality AND improvement AND competency | <https://www.google.com/search?q=healthcare+professional+AND+quality+AND+improvement+AND+competency&sca_esv=588229662&rlz=1C1GCEU_enMY943MY943&sxsrf=ACQVn09d7ycGVmjiw88P-kkAssy-THleAQ%3A1713768382385&ei=vgcmZpTvFon5seMPvt2H2Ac&ved=0ahUKEwiUxqLdnNWFAxWJfGwGHb7uAXsQ4dUDCBE&uact=5&oq=healthcare+professional+AND+quality+AND+improvement+AND+competency&gs_lp=Egxnd3Mtd2l6LXNlcnAiQmhlYWx0aGNhcmUgcHJvZmVzc2lvbmFsIEFORCBxdWFsaXR5IEFORCBpbXByb3ZlbWVudCBBTkQgY29tcGV0ZW5jeTIIECEYoAEYwwQyCBAhGKABGMMEMggQIRigARjDBEiMOVDeBljFGHABeAGQAQCYAbYBoAHeBaoBAzIuNLgBA8gBAPgBAZgCBqACywTCAgoQABiwAxjWBBhHwgIKECEYoAEYwwQYCpgDAIgGAZAGCJIHAzMuM6AHpjQ&sclient=gws-wiz-serp#ip=1> |
