# Supplementary material for: Quality Improvement Competencies for Health Care Quality Professionals: Protocol for a Scoping Review
Source: JMIR Res Protoc. 2026 Jun 4;15:e88787. doi: 10.2196/88787 (PMC13280533; doi:10.2196/88787)
Supplement: Multimedia Appendix 3 [file resprot_v15i1e88787_app3.docx]

Appendix 3: Data extraction form

| **Study Summary** | |
| --- | --- |
| Author |  |
| Publication Year |  |
| Publication Title |  |
| Article sources (Database/Grey Literature) |  |
| Origin/Country |  |
| Aim/purpose |  |
| Study design |  |
| Target population |  |
| Settings |  |
| Level of expertise (basic, intermediate, advance) |  |
| **QI Competencies** | |
| Domain (A domain is a comprehensive classification within a competency. It is a foundational ability that represents the required elements in the competency. Example: “Safety” or “Leadership”) | Sub-domain (A subdomain is a more specific sub-division within a domain. It breaks down the broader domain into more detailed components or specialised competence areas. Example: If the domain is “Safety”, the subdomain could be “Error Management” or “Patient Safety Principles”.) |
